# Is the Retina a Mirror of the Aging Brain? Aging of Neural Retina Layers and Primary Visual Cortex Across the Lifespan

**DOI:** 10.3389/fnagi.2019.00360

**Published:** 2020-01-08

**Authors:** Lília Jorge, Nádia Canário, Hugo Quental, Rui Bernardes, Miguel Castelo-Branco

**Affiliations:** ^1^Coimbra Institute for Biomedical Imaging and Translational Research (CIBIT), University of Coimbra, Coimbra, Portugal; ^2^Institute for Nuclear Sciences Applied to Health (ICNAS), University of Coimbra, Coimbra, Portugal; ^3^Faculty of Medicine, University of Coimbra, Coimbra, Portugal

**Keywords:** healthy aging, optical coherence tomography (OCT), magnetic resonance imaging (MRI), primary visual cortex (BA17), retinal segmentation, ganglion cell layer (GCL), outer segment photoreceptor/RPE complex (OPR)

## Abstract

How aging concomitantly modulates the structural integrity of the brain and retina in healthy individuals remains an outstanding question. Given the strong bottom-up retinocortical connectivity, it is important to study how these structures co-evolve during healthy aging in order to unravel mechanisms that may affect the physiological integrity of both structures. For the 56 participants in the study, primary visual cortex (BA17), as well as frontal, parietal and temporal regions thicknesses were measured in T1-weighted magnetic resonance imaging (MRI), and retinal macular thickness (10 neuroretinal layers) was measured by optical coherence tomography (OCT) imaging. We investigated the statistical association of these measures and their age dependence. We found an age-related decay of primary visual cortical thickness that was significantly correlated with a decrease in global and multiple layer retinal thicknesses. The atrophy of both structures might jointly account for the decline of various visual capacities that accompany the aging process. Furthermore, associations with other cortical regions suggest that retinal status may index cortical integrity in general.

## Introduction

The primary visual cortex, Broadman area 17 (BA17), receives direct sensory input through the lateral geniculate nucleus (LGN), conveyed from the retina and is the cortical area where retinotopic information processing begins. This region may undergo considerable neurodevelopmental reorganization in response to retinal input changes (d’Almeida et al., 2013; Ferreira et al., 2017).

Concerning retinocortical changes in response to disease-related degeneration, Barcella et al. (2010) resorting to voxel-based morphometry (VBM) demonstrated that patients with clinically installed Leber Hereditary Optic Neuropathy (LOHN)—marked by a predominant central macular ganglion cell degeneration and death—had reduced gray matter volume in the respective lesion projection zones of the primary visual cortex, compared to healthy controls. In fact, alterations in the thickness of this region are already present in asymptomatic patients suffering from the same disease, although in early stages changes are still compensatory (increased thickness in extrastriate visual cortex; d’Almeida et al., 2013). Moreover, also age-related retinal diseases, such as late-stage macular degeneration have an impact on cortical morphology. For example, Burge et al. (2016) found that the regions of the striate cortex that usually sample the visual input from the injured area of the retina were thinner in participants with macular disease when compared to controls. On the contrary, regions corresponding to non-damaged areas demonstrated a significant increase in cortical thickness. Overall, these findings suggest a strong retinocortical coupling of structural and functional changes in retinal disorders.

Healthy aging is characterized by diverse structural changes in both brain and retina, which association remains to be studied. Concerning the former, there is substantial evidence suggesting that widespread cortical shrinkage takes place with increasing aging (Courchesne et al., 2000; Good et al., 2001; Lemaître et al., 2005). The nature of such effects in early visual areas remains controversial. Whereas some studies have shown volume loss or cortical thinning of visual cortices (Resnick et al., 2003; Salat et al., 2004; Fjell et al., 2009), others have reported a certain sparing of these areas during aging (Allen et al., 2005; Grieve et al., 2005; Raz et al., 2005). Specifically to the primary visual cortex, according to Griffis et al. (2016), such discrepancy in the literature could be explained by methodological issues, since they demonstrated that different sub-regions of primary visual cortex were unequally affected by aging, depending on their retinotopic eccentricity. Nonetheless, studies directly examining the structure and function of primary visual areas are scarce in healthy aging, in spite of the fact that the organization of early visual areas is well documented in young adults.

Moreover, some studies have suggested that cortical alterations might contribute to age-related visual deficits, but the lack of studies on the visual cortex in healthy old individuals makes it difficult to know what changes actually occur.

The retina also undergoes substantial modifications throughout the lifespan. Histological studies have reported a reduction in the density of photoreceptors, ganglion cells, and pigment epithelial cells with age (Dolman et al., 1980; Quigley et al., 1989). Overall, retinal thickness studies using optical coherence tomography (OCT) imaging have revealed regional age-dependent differences in global macular integrity (Alamouti and Funk, 2003; Eriksson and Alm, 2009; Neuville et al., 2009; Sung et al., 2009; Song et al., 2010). The improvement of OCT image processing techniques has now allowed for the automatic segmentation of retinal individual layers, which provides a more detailed and specific perspective on such alterations. For instance, Demirkaya et al. (2013) measured the mean thickness of seven macular layers across different regions in subjects with different ages, and they found that the thickness of the pericentral ganglion cell layer (GCL), peripheral inner plexiform layer (IPL) and foveal outer segment layer (OS) were negatively correlated with age. Conversely, foveal retinal pigment epithelium (RPE) thickness was positively correlated with age.

The study of Won et al. (2016) addressed nine retinal layers and also found evidence of age-related morphologic changes. They compared a younger (<30) vs. an older group (>60) and found that the thickness and volume of peripheral retinal nuclear fiber layer (RNFL), GCL, and pericentral and peripheral IPL were superior in the first group, whereas the thickness and volume of foveal inner nuclear layer (INL) and inner retina (IR) were higher in the older group.

Furthermore, retinal alterations have been reported in patients with neurological disorders such as Stroke, Multiple Sclerosis, Parkinson and Alzheimer diseases (Grzybowski and Barboni, 2016; Alves et al., 2018). On the other hand, Satue et al. (2016) demonstrated that Alzheimer’s disease (AD) might share degenerative mechanisms that may be difficult to disentangle from ocular diseases, as for example glaucoma and age-related macular degeneration.

Given that the retina exhibits similarities to the brain in terms of functional, anatomic and immunological properties (London et al., 2013), it is not surprising that common degenerative mechanisms might affect both in health and disease. Consequently, there is a strong need to investigate the interdependence of both the brain and retina during healthy aging. This will also facilitate the identification of specific alterations resulting from age-dependent pathological mechanisms.

Thus, despite previous work on the impact of aging on the anatomic-physiology of each structure individually (retina and primary visual cortex), as far as we know, no efforts were made in order to investigate their integrity in the same study simultaneously. This is critical to directly investigate their concomitant aging. Therefore, we aimed to investigate the association of retinal layer and cortical integrity, in a healthy cohort aged between 20–80 years old. To that end, we performed MR structural data imaging measurements of cortical thickness in the primary visual cortex—BA17—the cortical area that receives direct retinal input—and OCT to measure the thickness of the macula and their individual layers, in the same set of participants. In parallel, we have also searched for correlations between the retina and other cortical regions—frontal, temporal and parietal lobes—in order to investigate whether associations between brain and retina go beyond the occipital lobe.

## Materials and Methods

### Participants

MR structural data acquisitions were obtained from 56 normally sighted controls (28 females, 28 males; with age ranging from 23 to 79 years old) without cognitive impairment, as assessed by the Montreal Cognitive Assessment (MoCA; Freitas et al., 2011) and with no clinical history of psychiatric or neurological disorders.

After excluding the history of any ocular disease or ocular clinical intervention, all participants underwent a comprehensive ophthalmological examination to check the absence of any visual complication, which included: visual acuity assessment with Snellen chart, ocular tension (Goldmann applanation tonometer), slit-lamp biomicroscopy and OCT imaging. Only subjects with normal or corrected to normal vision (visual acuity ≥ 8/10) were included, with the refractive error between ± 5 diopters, and with intraocular pressure ≤21 mmHg.

Images of the retina were obtained from the same set of participants with spectral-domain OCT (Cirrus HD-OCT 5000, Carl Zeiss Meditec, Dublin, CA, USA) and only participants without any abnormalities of the optic disc or macula were included. Additionally, we used as exclusion criteria family history of glaucoma, or any other hereditary eye disease; diabetes, because conditions of the vascular system may concomitantly impact on cortical and retinal health (Patton et al., 2005) or other systemic disease that could affect the eye.

The study was approved by the Ethics Committee of the University of Coimbra, and all subjects participated voluntarily and gave their informed written consent for the study, according to the Declaration of Helsinki.

### Retinal Imaging

For each participant, the retinal images from both eyes were acquired using OCT resorting to the HD-OCT Cirrus 5000 system with the macular cube 512 × 128 protocol. This protocol creates a cube of data through a 6 mm square grid around the fovea centralis by acquiring a series of 128 horizontal B-scans lines each composed of 512 A-scans, with an axial resolution of 5 μm. Two experienced technicians performed all the acquisitions.

### MRI Imaging

Magnetic resonance imaging (MRI) data were acquired in a 3 Tesla Siemens Magnetom Trio Scanner, using a whole-brain approach, with a 12-channel head coil. Two 3D anatomical MPRAGE (rapid gradient-echo) T1-weighted sequence were acquired with the following acquisition parameters: 1.0 × 1.0 × 1.0 mm^3^ voxel resolution, repetition time (TR) 2,530 ms, echo time (TE) 3.42 ms, and a field of view (FOV) of 256 × 256 mm. The anatomical sequence comprised 176 slices, a flip angle of 7° and an inversion time of 1,100 ms.

#### MRI Data Analysis

Brain image processing was conducted using BrainVoyager QX software (version 2.8.2), which allows largely automatically cortex segmentation through the application of a set of advanced tools, and reliable cortical thickness measurements resorting to the second-order partial differential Laplace’s equation. To each anatomic data set, the following pre-processing steps were executed in all subjects: a bias-field mask (Dale et al., 1999) was applied in order to diminish the intensity variations caused by magnetic field and RF-field inhomogeneities, before the inhomogeneity correction a mask was applied to extract the skull and remove the extra-cerebral voxels (Goebel et al., 2006). The two structural data acquisitions of each subject were then averaged, to increase the signal-to-noise ratio, re-oriented into the AC-PC plane and then transformed into the Talairach reference system. Later on, the anatomical data were submitted through the advanced automatic segmentation routines for later cortical thickness measures. Simultaneously, the same anatomical volumes were segmented using automatic routines (Kriegeskorte and Goebel, 2001) to create surface representations (meshes) of each hemisphere for further measurements on surface space.

#### Cortical Thickness Assessment

Cortical thickness measurements were obtained using the BrainVoyager software pipeline, which starts with the automatic cortex segmentation in volume space of white matter-gray matter (WM-GM) and gray matter-cerebrospinal fluid (GM-CSF) boundaries. In a preliminary stage, the anatomical images were transformed to 0.5 × 0.5 × 0.5 mm iso-voxels using sync interpolation. Thereon, the ventricles and subcortical structures were masked and, as the final preparatory step, a sigma filter was used to enhance the contrast between the GM and WM tissue types.

Thereafter, an adaptive region growing step relying on locally computed intensity histograms and gradient information was used to split white from gray matter voxels. The resulting noisy WM-GM border was smoothed by computing a magnitude map build upon computed gradient maps of the binary segmentation outcomes. After that, a dilatation process starting at the WM-GM border enabled the CM-CSF border segmentation.

At the end of the automatic segmentation process, a dataset consisting of two intensity values was obtained, one representing the WM and the other the GM. This dataset was then used for the cortical thickness measurements. The software uses an automatic algorithm that applies the second-order partial differential Laplace’s equation, as described in Jones et al. (2000). After the thickness calculation, a cortical thickness map was shown overlaying on the volumetric data set (VMR).

Since we aimed to compare the thickness values from a specific area, the primary visual area BA17, between all subjects and examine aging effects, it was necessary to guarantee an exact correspondence of this area among the different participants. Since the Talairach normalization produces only a rough alignment between subject’s cortices, the subsequent analyses were conducted in the surface space (previously created cortical meshes) in order to benefit from the cortex-based alignment (CBA), as described in Geuze et al. (2008), of the different subject’s brains.

**Figure 1:**
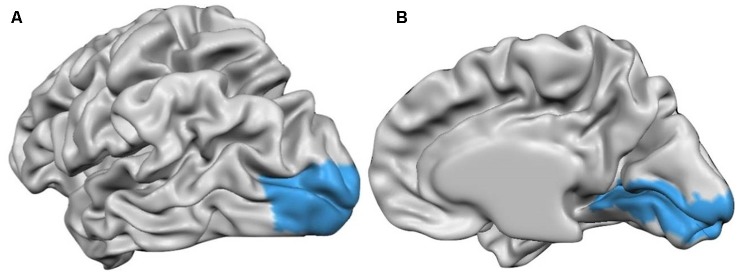
Cortex surface view of the anatomically outlined BA17 used to measure the cortical thickness [**(A)** left hemisphere **(B)** right hemisphere].

Thus, after the cortical measurements in volume space, the volumetric maps were interpolated to the individual cortex meshes that resulted from a former CBA, creating the subsequent surface cortical maps (SMPs). After the SMPs were made to all subjects, they were provided to the software along with a Patch-Of-Interest (POI) file containing the BA17 surface patch (Figure 1), and the information describing the alignment of the individual cortex to the target cortex. Additionally, we have also measured the thickness values from frontal, temporal and parietal lobes following the same methodology. Thus, we provided to the software the POIs files containing the frontal, temporal and parietal anatomically delineated regions (BV mask) along with the information describing the alignment of the individual cortex to the target cortex, for thickness measurements inside these regions.

Afterward, the thickness values averaged over all POIs vertices for each subject were calculated for the right and left hemisphere individually, and exported for further statistical analyses on SPSS software.

### Retinal Thickness Assessment

For each participant’s eye, the OCT acquisition went through an automatic segmentation routine resorting to the *Iowa Reference Algorithms* (Retinal Image Analysis Lab, Iowa Institute for Biomedical Imaging, Iowa City, IA, USA; Li et al., 2006; Garvin et al., 2009; Abràmoff et al., 2010) that allows significantly accurate segmentation up to 10 retinal layers.

**Figure 2:**
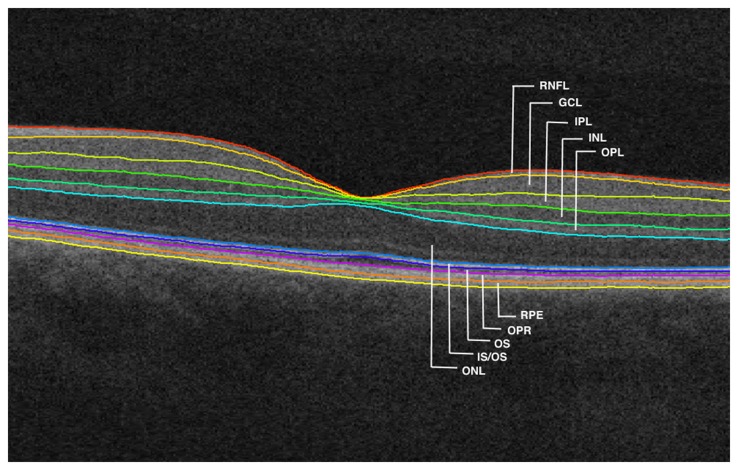
Segmented retinal layers: retinal nerve fiber layer (RNFL); ganglion cell layer (GCL); inner plexiform layer (IPL); inner nuclear layer (INL); outer plexiform layer (OPL); outer nuclear layer (ONL); inner segment/outer segment junction (IS/OS); outer segment (OS); outer segment photoreceptor/RPE complex (OPR); retinal pigment epithelium (RPE).

From each macular volume, 11 surfaces, outlining the 10 retinal layers, were automatically segmented by the algorithm mentioned above. Consequently, the layers investigated in this study were: RNFL; GCL; IPL; INL; outer plexiform layer (OPL); outer nuclear layer (ONL); inner segment/outer segment junction (IS/OS); outer segment (OS); outer segment photoreceptor/RPE complex (OPR), retinal pigment epithelium (RPE), see Figure 2. All B-scans and surface layers were visually assessed, and manual corrections on the segmentation were introduced wherever required. The thickness of each layer was computed as the distance (in voxels) between the two surfaces delimiting the layer, multiplied by the voxel size in that direction. This value was provided by the segmentation software based on the number of voxels and imaging depth.

The thickness of the all lamina (global retinal thickness), as well as of each macular layer, were calculated as the average from the complete macular area.

### Statistical Analyses

Overall, 56 subjects were considered for cortical thickness analysis and only 52 were considered for the retinal thickness analysis (four OCT acquisitions were excluded due to acquisition problems). Brain/retina correlation analyses were consequently limited to 52 cases.

The mean cortical thickness values of BA17, frontal, temporal and parietal regions were obtained for each participant calculated as the average of all vertices inside these regions, and a mean value from both hemispheres was calculated for each participant.

Concerning the retina, the mean thickness value was calculated for each retinal layer and all lamina (global retinal thickness), by averaging the values from both eyes to each individual.

Pearson correlation coefficients were computed between age and thickness measurements to investigate the relationship of both structures with age, as well as between BA17 and all lamina thicknesses to study the interplay between both. Additionally, a multivariate ANOVA (MANOVA) analysis including the 10 segmented layers followed by *post hoc* univariate analysis was performed to study the individual behavior of each layer with age.

Thereafter, a multiple regression analysis was run to inspect whether retina integrity indices and age can predict cortical integrity, to further assess the relationship between the retina and cortex integrity.

In a final analysis, we also searched for associations between other cerebral areas—frontal, temporal and parietal areas—and retina.

Data analysis was conducted in IBM SPSS Statistics (version 22.0), and for graphs, GraphPad Prism (version 6.0) was used. The tests were performed two-tailed and *p* < 0.05 was used as a threshold for statistical significance.

## Results

For the cortical measurements, 56 subjects were considered, gender-balanced, with age ranging from 23 and 79 years, and average ± standard deviation (SD) of 47.03 ± 16.89 years (see Table 1).

**Table 1:** Age distribution of the participants included in the study and the number of smokers by group of ages.

Age range	Participants (*n* = 56)	Light Smokers
20–29	11	3
30–39	9	1
40–49	10	2
50–59	10	3
60–69	10	0
70–79	6	0

Regarding the macular measurements, 52 subjects were considered, also gender-balanced, with age ranging from 23 to 79 years, as above, and average ± SD of 46.77 ± 17.27 years.

**Figure 3:**
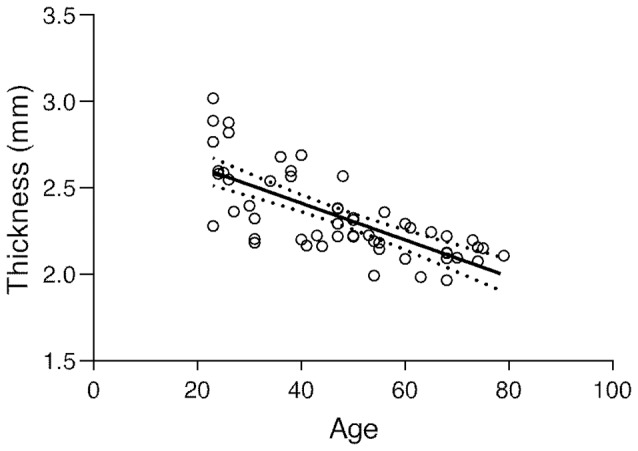
Regression plot of the BA17 thickness vs. age and 95% confidence of the regression slope (*Y* = −0.01062 × *X* + 2.836; *r* = −0.724; *p* < 0.0001).

The subject’s average ± SD of BA17 cortical thickness was 2.337 ± 0.246 mm. The corresponding regression plot is depicted in Figure 3. Simultaneously the correlation test demonstrated a strong negative statistically significant correlation between age and BA17 average cortical thickness (*r* = −0.724, *p* < 0.0001), showing that the primary visual cortex becomes significantly thinner as people get older (about 106 μm per decade, based on the regression equation).

**Figure 4:**
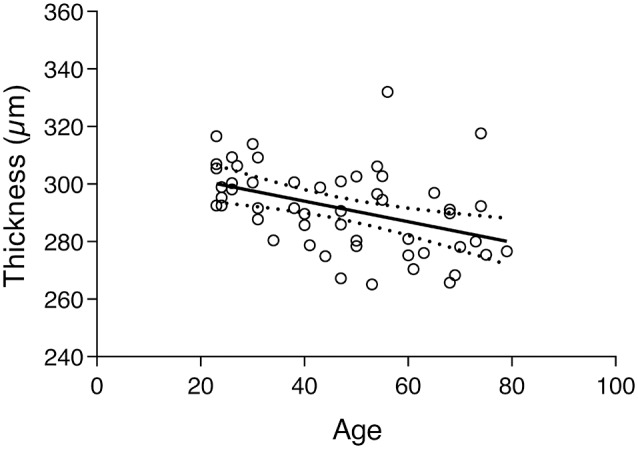
Regression plot of the total retinal thickness vs. age and 95% confidence of the regression slop (*Y* = −0.3566 × *X* + 308.3; *r* = −0.4186; *p* < 0.0020).

The subject’s global thickness average was 291.6 ± 14.71 μm. A similar finding as above was found between the global thickness and age since the correlation test shows a statistically significant negative association between the two variables (*r* = −0.4186, *p* < 0.0020). The correspondent regression plot can be seen in Figure 4. The decrease of macular thickness with age is 3.6 μm per decade.

To investigate general and specific effects at the retinal level, we have computed a multivariate ANOVA (MANOVA), using age as a predictor and the individual layers as dependent variables. Overall we found that age significantly predicts the macular thickness, Pillai’s Trace = 0.456, *F*_(10,41)_ = 3.44, *p* = 0.002. *Post hoc* analysis showed that GCL: *F*_(1)_ = 9.483, *p* = 0.003; IPL: *F*_(1)_ = 8.453, *p* = 0.005; INL: *F*_(1)_ = 9.218, *p* = 0.004; ONL: *F*_(1)_ = 7.813, *p* = 0.007; OS: *F*_(1)_ = 8.970, *p* < 0.004 and OPR: *F*_(1)_ = 14.54, *p* < 0.001 were significantly predicted by age.

**Figure 5:**
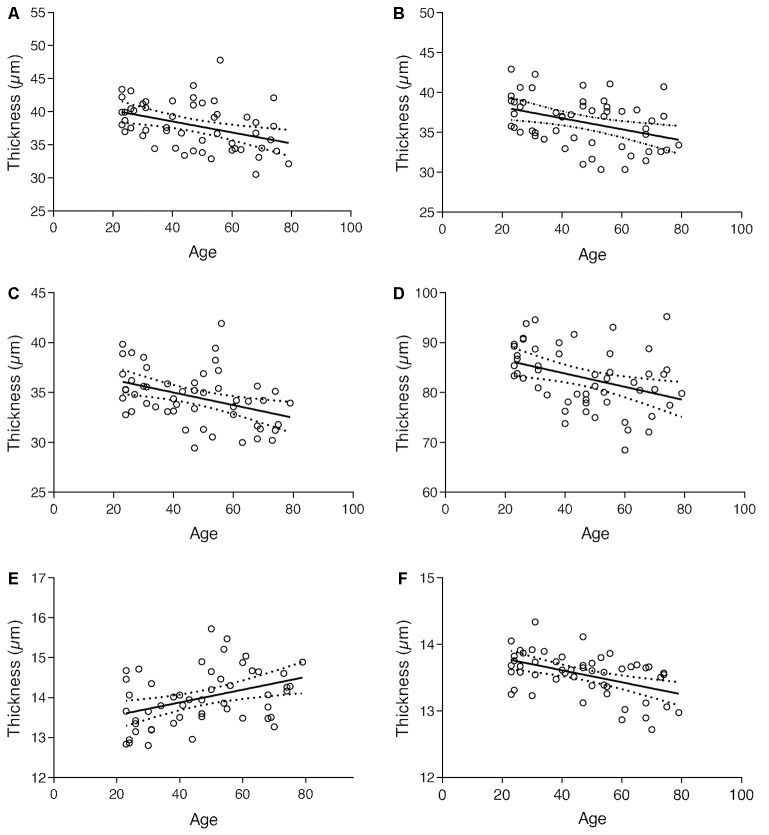
Regression plot of **(A)** GCL; **(B)** IPL; **(C)** INL; **(D)** ONL; **(E)** OS and **(F)** OPR thickness vs. age and 95% confidence intervals for the regression line.

Plots in Figure 5, along with results from the multivariate analysis, show that the thickness of the GCL, IPL, INL, ONL, and OPR layers decreased with age, whereas the thickness of OS layer increased. The RNFL, OPL, IS/OS, and RPE layers, on its turn, showed no significant alterations with age.

**Figure 6:**
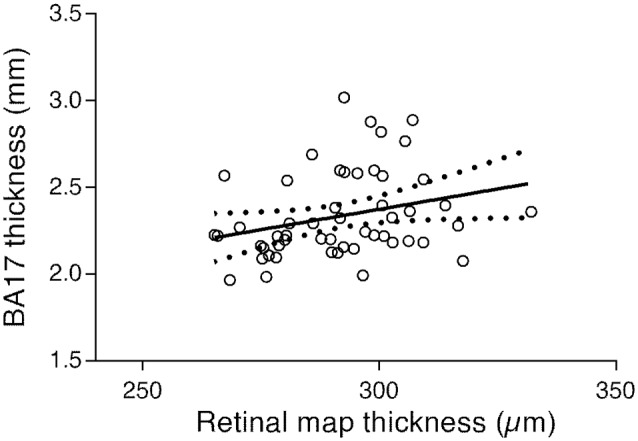
Regression plot of the BA17 thickness vs. all lamina macular thickness and 95% confidence of the regression slope (*Y* = 0.004687 × *X* + 0.9676; *r* = 0.277; *p* < 0.047).

The results from the correlation tests performed to measure the association between cortical and retinal thickness showed a positive association between BA17 and the overall thickness of all lamina (*r* = 0.277, *p* < 0.047). The respective regression plot is presented in Figure 6, showing that the primary visual cortex is positively associated with macular thickness; a decrease of macular thickness is significantly associated with a decrease in BA17 thickness (about 0.0047 mm per unit of macular thickness change, according to the regression equation).

Considering the association between global macular thickness and BA17, we have posteriorly run a multiple linear regression analysis to verify whether age and all lamina thickness are potentially predictive of BA17’s thickness. These variables statistically significantly predicted BA17, *F*_(2,49)_ = 27.018, *p* < 0.0001, *R*^2^ = 0.52. Age was the only variable that contributed significantly to the prediction ($\reta$ = −0.737, *p* < 0.0001) Thus, when adjusting for age we can see that the relation between cortex and retina is disrupted. Therefore, we can conclude that age is the main responsible for the association between the two structures.

Considering the emerging literature relating GC-IPL to putative cortical integrity we have also computed the correlation with GC-IPL and results show a significant positive correlation between both (*r* = 0.300, *p* < 0.030).

We have further computed a multiple linear regression, using all the individual layers and age as predictors for BA17 integrity.

The results have shown that these variables statistically significantly predicted the interaction between age and BA17 *F*_(11,40)_ = 6.438, *p* < 0.0001, *R*^2^ = 0.6391. We found that OPR ($\reta$ = −0.272, *p* < 0.05) and age ($\reta$ = −0.895, *p* < 0.0001) were the only variables that significantly interact to explain the variance of BA17 thickness. Thus, we can conclude that age *per se* is the main responsible for the interplay between cortex and retina.

In addition, correlations between other cortical regions and retina showed a significant association between frontal (*r* = 0.351, *p* = 0.011), parietal (*r* = 0.350, *p* = 0.011) and temporal (*r* = 0.335, *p* = 0.015) lobes. Therefore, this link between brain and retina might not be limited to the occipital region, suggesting that retinal thickness may be indicative or an accessible read-out of cortical structure in general.

## Discussion

This study is, to the best of our knowledge, the first that attempted to evaluate, in the same sample of healthy individuals, structural changes in both cortex and retina as a function of age in anatomically defined primary visual cortex (BA17) and in 10 different macular layers, and its associations. Our results provide evidence of a positive relationship between the thicknesses of these structures and a concomitant negative correlation with age. While it is in general well accepted that a heterogeneous global age-related loss of cortical tissue occurs, results across studies regarding the primary visual cortex have been inconsistent. Nonetheless, several groups have indeed reported a decrease in gray matter thickness of this region, as we found in the present study. For instance Salat et al. (2004), Fjell et al. (2009), Ziegler et al. (2010) and McGinnis et al. (2011), who performed whole-brain approaches measures, that model the impact of aging at each surface vertex, found evidence of an age-related decline in primary visual cortex thickness. Brewer and Barton (2016) found that healthy aging subjects do have the reduced surface area and increased pRF sizes in the foveal representations (0° to 3° eccentricity) of V1 (primary visual cortex) as compared with healthy young control subjects. Moreover, Sowell et al. (2003) suggested that occipital cortex surrounding the calcarine sulcus exhibits a linear pattern of gray matter density loss with age.

It is worth to mention that our results are not consistent with some whole-brain studies suggesting a certain sparing of the occipital cortex during the aging process when compared to parietal-temporal regions (Thambisetty et al., 2010) since we found evident atrophy of BA17 across age. However, our results might be explained by the fact that we have conducted a region-based analysis in a relatively large sample.

On its turn, Lemaitre et al. (2012) in a whole-brain approach did not find significant age-related reduction in volume, thickness or surface area in the occipital cortex. They pointed out as a possible explanation for their results, the fact of their sample had only 17% of individuals with age above 60 years, and they hypothesized that it is above that age that this area possible suffers a more significant reduction. In fact, McGinnis et al. (2011) found that thinning in the primary visual cortex was highest in their older sample. Raz et al. (2004), who traced ROIs manually, did not find age-related differences in the volume of this region. Likewise, Crossland et al. (2008) found no changes in the overall size of V1.

Despite the incongruities in the existing literature, overall, our data corroborate the notion that this visual area does become thinner with advancing aging. Nevertheless, such discrepancies between studies could derive from differences in analytic techniques, where measures of cortical thickness and volume are detecting different degenerative processes with distinct sensitivity (Ziegler et al., 2010), differences in the population characteristics (Lemaitre et al., 2012), or in the way that this region is defined (Griffis et al., 2016). In the present study, and in addition to a well-distributed sample across ages, we resorted to surface-based thickness tools, known to be more sensitive for the detection of age-related morphological decline (Hutton et al., 2009).

Concerning the retina, during lifetime the macula undergoes significant degenerative changes, causing visual decline and often visual loss. In a healthy population, our data revealed a significant decrease in the total macular thickness with increasing age. Overall, there is a broad consensus about retinal loss with age (Alamouti and Funk, 2003; Eriksson and Alm, 2009; Sung et al., 2009; Casaletto et al., 2017), possibly resulting from the loss of retinal cells such as bipolar, ganglion cells, as well as photoreceptor cells (Gao and Hollyfield, 1992; Curcio et al., 1993; Harman et al., 2000; Aggarwal et al., 2007). Moreover, vascular compartments may also decrease over time (Klein, 2006; Sun et al., 2008).

Imaging studies with the segmentation of retinal layers are scarce. To the best of our knowledge, this is the first study to differentiate the retina in 10 distinct layers addressing the effect of aging on thickness. In the previous study of Won et al. (2016) (only addressing the retina and not the cortex), nine retinal layers were individualized using the same algorithm as ours.

Our measurement outcomes revealed that while some layers remained stable with age (RNFL, OPL, IS/OS, and RPE), others decreased (GCL, IPL, INL, ONL, and OPR), and only one of them appeared to be increased in terms of thickness (OS). In particular, our results demonstrated a GCL age-related thinning, similarly to other findings (Ooto et al., 2011; Demirkaya et al., 2013; Won et al., 2016). In fact, this layer seems to be one of the most prone to be affected by aging mechanisms, being in agreement a significant decline of the number of ganglion cells across age (Balazsi et al., 1984; Gao and Hollyfield, 1992; Harman et al., 2000; Kerrigan-Baumrind et al., 2000; Nag and Wadhwa, 2012).

We also found evidence of IPL and INL reduction being in accordance with previous studies (Ooto et al., 2011; Demirkaya et al., 2013; Won et al., 2016). Evidence about how INL cells change with age and respond to different pathological conditions is scarce (Nag and Wadhwa, 2012). This decrease could, however, result from the loss of some neurons or glial cells, as it is known to occur with bipolar cells (Aggarwal et al., 2007). Regarding IPL, since it comprises synapses between the amacrine and bipolar cells with ganglion cells dendrites, it seems feasible that the shrinkage of this layer could be attributed to a loss of both bipolar and ganglion synapses. The studies of Demirkaya et al. (2013) and Ooto et al. (2011) also found an age-related decrease of this layer across age.

Likewise, ONL and OPR showed to be affected negatively by aging. Gartner and Henkind (1981) were the first to report a thinning of the ONL with aging, as a result of photoreceptor loss. Subsequent studies have shown substantial loss of rods, but not cones, from the periphery (Gao and Hollyfield, 1992) and macula with aging (Curcio et al., 1993; Jackson et al., 2002). This age-related photoreceptor loss might also contribute to the OPR thinning, as found in the current study.

Conversely, RNFL, OPL, IS/OS, and RPE did not show differences with our morphologic metrics with increasing age. Despite the considerable evidence pointing out to a decrease in retinal neuron fibers, resulting from ganglion cell loss (Balazsi et al., 1984; Harman et al., 2000; Kerrigan-Baumrind et al., 2000; Nag and Wadhwa, 2012), the majority of the studies have focused on peripapillary measures, around the optic disk, rather than on macular RNFL (Alamouti and Funk, 2003; Eriksson and Alm, 2009; Sung et al., 2009). For instance, Demirkaya et al. (2013) indeed found a thinning in peripapillary RNFL, but their results did not reveal a significant decrease in the whole macular RNFL thickness. Instead, a slight increase was verified in the pericentral ring. Won et al. (2016), in turn, when comparing a young group with an older one, found a significantly lower thickness and volume in the RNFL peripheral region in the latter. Also, Neuville et al. (2009) have reported only an age-related decreasing of this layer thickness in the more peripheral nasal region. Thus, one might assume that an age-related decrease probably occurs, however just detectable in regions where the axons are denser, yet when the full thickness is analyzed this subtle effect might become undetected.

The fact that may explain the consistently reported decrease of axons in the region of the optic disc is that it corresponds to the region where axons of the GCL converge, including those from the extramacular area. As such, it is not surprising that this region presents a higher GCL decrease (Harman et al., 2000) compared with the macular area. Nonetheless, Ooto et al. (2011) found a significant decrease in the RNFL thickness in the macula.

Concerning the OPL, our results are in line with the works of Ooto et al. (2011), Demirkaya et al. (2013), and Won et al. (2016), who studied the same layer in their sample. With regard to the RPE, it undergoes considerable changes through age, including loss of melanin granules, accumulation of lipofuscin, basal deposits, and thickened Bruch’s membrane (Bonilha, 2008). As a result, there are claims that the RPE thickens with age, at least in its central area (Staurenghi et al., 2014). However, and in accordance with Demirkaya et al.’s (2013) and Won et al.’s (2016) results, we did not find an increase in the total macular RPE thickness although we do find the change mentioned above in the OS, which is tightly related with age-related changes in RPE. Thus, it seems likely that the thickness of this layer might increase with age only in specific sub-regions, namely in the more central macula. For example, Demirkaya et al.’s (2013) results show that as a whole the RPE thickness did not change, but increased significantly in the foveal region. Besides, there are arguments about a reduction in the number of pigment epithelium cells (Gao and Hollyfield, 1992; Watzke et al., 1993; Panda-Jonas et al., 1996), which might balance the cell deposits, perhaps explaining the absence of structural changes in our results. Similarly, Won et al. (2016) did not find differences between groups (younger and adults) in the RPE in any of the regions analyzed.

Results pertaining to the only macular layer that revealed increased with age, that is, the OS, are in agreement with Ooto et al. (2011). One should note, however, that their OS definition also included the RPE, precluding direct comparisons. These structures show an intimate functional relationship. Moreover, segmentation beyond the ONL, in the outer macula, has been more uneven across studies. The OS is known to be the locus of high membrane turnover with the accumulation of its products as aging progress, which might explain its increases with age. Thus, we do believe that a higher OS thickness could derive from the excessive metabolic strain that accumulates with aging. In fact, the RPE is responsible for the OS phagocytosis (Hogan et al., 1974; Anderson et al., 1978; Bok, 1985; Holtkamp et al., 2001; Strauss, 2005), a process that is known to deteriorate with age (Dorey et al., 1989; Bonnel et al., 2003).

In convergence with the concept that the primary visual cortex goes through considerable modifications in response to retinal changes, in the current work, we were able to show that aging *per se* is associated with such effect. When a multivariate analysis was carried out, our results showed that apparently, OPR is the only layer that explains the variance of BA17 thickness through an interaction term, e.g., changes are not solely justified by age. The atrophy of both structures might account for the decline of various visual capacities that accompany the aging process. For instance, Weale (1975, 1978, 1982) suggested that the decline in acuity results from a random loss of neurons in the retina or in central visual pathways. In turn, Brewer and Barton (2016) suggest that the functionally detected alterations in PRF’s and visual field maps as well as surface reduction in foveal V1 might account for the age-related alterations in vision such as reduced visual acuity and decreased contrast sensitivity at medium and high spatial frequencies.

In fact, normal aging leads to a decline of several visual abilities that cannot be attributed only to optical changes, and also derive from neuronal changes in the retina and/or brain (Elliott, 1987; Whitaker and Elliott, 1992; Spear, 1993). Nevertheless, the relative roles of each one are still unclear. For example, while some researchers have suggested that the decline in spatial-frequency contrast sensitivity is due to retinal changes (Sloane et al., 1988), others have related it to cortical mechanisms (Crassini et al., 1988). Thus, our results shed some light on this issue, since they demonstrate that there are morphologic changes in both structures, which probably account for the loss of visual functions that have been repeatedly reported by psychophysical studies studying a broad range of visual channels (Mateus et al., 2013; Silva et al., 2014). Such age-related changes in healthy population are of critical importance to understand the ones emerging in pathological neurodegeneration (Silva et al., 2005; Castelo-Branco et al., 2009; Lemos et al., 2012, 2016; Graewe et al., 2013), including regions beyond visual areas, as demonstrated here.

Despite the focus of the present work on studying the impact of age in the visual system, by assessing the structural integrity of both retina and primary visual cortex (the cortical area that receives direct projections from the retina) and their relationship, other regions were also investigated. We have therefore additionally searched for associations between the retina and other cortical regions—frontal, parietal and temporal lobes.

In fact, recent reports have suggested that retina is an approachable window to neurodegenerative diseases (Yu et al., 2014; Thomson et al., 2015; den Haan et al., 2018) and in healthy brain aging (Liu et al., 2016; Casaletto et al., 2017). Accordingly, we identified that associations between retina and brain exist beyond the visual areas. This finding suggests that the retina indexes cerebral cortical changes irrespective of retinocortical connectivity. Brain and retina, since their similarities, might share the same neurodegenerative mechanisms that occur in parallel across the lifetime.

Other studies had also searched for associations between the retina and cortical integrity in healthy individuals. For instance, Casaletto et al. (2017) in neurologically normal older adults, have found evidence of an association between medial temporal lobe volume (encompassing bilateral entorhinal, parahippocampal, and hippocampal volumes) with peripapillary RNFL thickness, macular volume, and macular ganglion cell volumes, while associations between other regions such as parietal-temporal regions, precuneus or basal ganglia were not found.

Liu et al. (2016), in a whole-brain approach, searched for associations between GCL-IPL and whole cortex white matter (WM) integrity as well as gray matter volumes (GMV) in three distinct groups of participants, a group of healthy individuals, a group of mild cognitive impairment subjects and other of AD patients. For subjects with no cognitive impairment (healthy aging), they found that a GC-IPL loss of integrity was related to a lower WM microstructure integrity in several regions as for example in frontal, parietal, and cingulum regions, and was weakly associated with GMV reduction in occipital lobe and cerebellum. Interestingly, this association was lost in the other two groups.

In addition, in a recent study, an association between a decrease in GC-IPL thickness and a decrease in GMV of the occipital and temporal lobes was found in an elderly heterogeneous sample composed by three demented subjects, 36 with no cognitive impairment (healthy subjects) and 125 with cognitive impairment (Ong et al., 2015). On its turn, den Haan et al. (2018) found a significant correlation between retinal thickness and cortical parietal atrophy in healthy controls and a cohort of AD patients, suggesting an association between the two structures that is independent of amyloid.

## Conclusion

The present study enables a better understanding of how the visual system ages, by providing concomitant measurements of neuroretinal layers and cortical thickness. We provide evidence suggesting that the primary visual cortex suffers an age-related loss that leads to its shrinkage, a finding that it is not consensual yet and is highly debated. Second, we demonstrate how 10 different individual layers anatomically change with increasing aging and identify novel and distinct patterns of change in different layers. Finally, associations between brain and retina were found beyond visual cortices (frontal, parietal and temporal regions), suggesting that retinal health may indeed be a potential read-out of cortical structure in general.

## Data Availability Statement

The datasets generated for this study will not be made publicly available. They are still being used for research with the appropriate consent forms, but they can be released at a later stage, or after a request to the corresponding author.

## Ethics Statement

The studies involving human participants were reviewed and approved by Comissão de Ética da Faculdade de Medicine da Universidade de Coimbra. The patients/participants provided their written informed consent to participate in this study, according to the Declaration of Helsinki.

## Author Contributions

LJ designed the study, performed data acquisition and analysis and wrote the article. NC performed data acquisition and analysis and revised the article. HQ performed data acquisition and analysis. RB performed data acquisition and analysis. MC-B designed the study, performed data analysis and wrote the article.

## Conflict of Interest

The authors declare that the research was conducted in the absence of any commercial or financial relationships that could be construed as a potential conflict of interest.
